# Prevalence and risk factors associated with *Taenia solium* cysticercosis in pigs in Oyam district, Uganda

**DOI:** 10.1371/journal.pntd.0013776

**Published:** 2025-12-02

**Authors:** Ronald Were, Alfonse Opio, Gabriel A. Kagenda, Geoffrey Maxwell Malinga, Harriet Angwech

**Affiliations:** 1 Department of Biology, Faculty of Science, Gulu University, Gulu, Uganda; 2 Production Department, Oyam District Local Government, Oyam, Uganda; Uniformed Services University: Uniformed Services University of the Health Sciences, UNITED STATES OF AMERICA

## Abstract

**Introduction:**

*Taenia solium* cysticercosis is a disease known as porcine cysticercosis (PC) in swine/pigs but also taeniosis and cysticercosis in humans. The larval stage of the pork tapeworm (*T. solium*) has for decades been responsible for lowering economic productivity of pigs and has direct human health defects. This study assessed the disease situation in pigs to provide baseline data for appropriate disease control in humans and pigs in Oyam district following a significant improvement in latrine coverage in Uganda.

**Methodology:**

A study was conducted in Oyam district in which blood was obtained from 394 pigs and analyzed for the presence or absence of circulating antigens of *T. solium* using a commercial enzyme-linked immunosorbent assay (cysticercosis Ag ELISA). The sampled pigs were also subjected to lingual examination for the presence or absence of *T. solium* cysts. Furthermore, structured questionnaires were administered to pig owners to collect information about pig management systems, feeding, housing, sex, breed and ages of pigs as well as awareness of *T. solium* cysticercosis and its control. The association of risk factors with prevalence of porcine cysticercosis was assessed using chi-square and logistic regression analyses at the 95% confidence level. These results were then compared with those of Nsadha et al. (2010).

**Principal findings:**

The overall prevalence of PC was 27% (Lingual examination) and 33% (ELISA), indicating a 17% increase in infection rates in the district from the result of Nsadha et al. (2010). While latrine coverage increased from below 50% to 74 an increase of 24%. Local breeds, age of pigs, poor household hygiene, consuming pork and tethering and free-range husbandry systems were significant predictors of infection. There was no significant association between the possession or use of latrine and prevalence of PC in the study area.

**Conclusions/significance:**

PC is still endemic in Oyam District and significant risk factors are: breeds, age, husbandry practices, pork consumption and household hygiene. Therefore, efforts to expand latrine coverage should be accompanied by improvements in latrine quality, community sensitization, and broader investments in sanitation infrastructure in the study area and in other settings with similar epidemiological profiles. Furthermore, future studies could factor in the impact of environmental contamination and latrine status to give a comprehensive picture of the epidemiology of the infection in the area.

## Author summary

Porcine cysticercosis, caused by the larval stage of the pork tapeworm (*Taenia solium*), is a zoonotic disease that affects both pigs and humans. In pigs, it reduces economic value, while in humans it can cause taeniosis and cysticercosis, including neurocysticercosis, a major cause of epilepsy in endemic regions. We conducted a cross-sectional study in Oyam District, northern Uganda, to update the status of the disease following improvements in latrine coverage. Blood samples from 394 pigs were tested for *T. solium* antigens, and pig owners were interviewed about pig management, hygiene, and knowledge of the disease. The prevalence of porcine cysticercosis was high, at 27% by lingual examination and 33% by ELISA—higher than previously reported in the area. Significant risk factors included pig breed, age, husbandry practices (tethering and free range), pork consumption, and poor household hygiene. Unexpectedly, latrine ownership and use were not associated with infection, suggesting that environmental contamination may play a greater role in sustaining transmission. These findings highlight the need for integrated control measures focusing on pig husbandry, hygiene, and community awareness, in addition to sanitation improvements, to reduce the burden of *T. solium* in both pigs and humans.

## 1. Introduction

Cysticercosis is an infection caused by a larval (intermediate) stage of the tapeworm *Taenia solium*. It is a zoonotic disease of significant public health and economic importance in Asia, Latin America and Sub-Saharan Africa [1,2]. In these regions, the interplay of poverty, insufficient education, restricted management capacity, limited access to diagnostic services and lack of efficient prevention and control measures contribute to the high endemicity [3]. Epidemiologically, the infection is most common in the pork consuming areas of the world [4] leading to considerable production, productivity, and fertility losses. A monetary loss of approximately €194 per case has been reported in countries where this disease is prevalent [1,5]. Among the thirteen endemic zoonoses known to cause most illnesses to livestock keepers, cysticercosis is ranked third [6] and yet considered to be a potentially eradicable disease by the World Health Organization [3]. Besides neurocysticercosis, in humans it is also responsible for acquired epilepsy [7,8].

Pork per capita consumption has more than doubled over the last 50 years as a result of urbanization, diet supplementation and income growth [6,9]. Uganda ranks highest in East Africa at 3.4 kg per person per year [10–12]. Furthermore, the Food and Agriculture Organization (2011) projects that Uganda’s overall pork consumption would rise by 184% between 2000 and 2030 as a result of the country’s growing population. We expect further increase in number of pigs too with the government interventions such as the Parish Development Model (PDM) as a good number of the beneficiaries have invested in piggery. This increase comes with increased risk of zoonotic disease transmission to pig farmers and consumers requiring continuous monitoring and surveillance of the infections in pig population.

Piggery farming in Uganda is a growing enterprise, with most pigs kept under free -range or tethering systems roaming in search of food [5]. These pigs end up in the local market, where they are purchased by butchers or traders directly from households [13]. The free-range system exposes pigs to contaminated materials such as human fecal matter and vegetation leading to PC [13], and consequently making the pork a potential threat to human health. By law, pork inspection is required at slaughter facilities in Uganda; however, this is rarely carried out due to the limited number of pork inspectors, allowing infected pork to enter the market [13]. In addition, most pigs are slaughtered in ungazetted areas and sold directly in informal markets without meat inspection [12]. This practice places pork consumers at a high risk of *T. solium* infection.

The major risk factors of PC are poor hygiene and sanitation among households, pig husbandry systems especially free-range system and lack of knowledge/awareness on *T. solium* cysticercosis and its control [14–16]. A recent health sector performance report of the Uganda Ministry of Health (AHSPR, 2022/23) indicates that only 44% of Ugandan households own and use improved toilet/latrines; 50% own/use unimproved open latrines and 5% of the households lack latrines while latrine coverage has risen to about 73% across the country. The report further notes that in some regions, e.g., the Lango sub-region, up to 14% of households lacked latrines and yet some of these (such as Oyam) are known to be among the leading pig keeping districts in the country. The hygiene and sanitary conditions as well as the change in production and consumption trends of pigs is anticipated to influence the epidemiology of *T. solium* cysticercosis in Uganda increasing the risk of further spreading the infection to both the pig and human population.

An earlier study in the Lake Kyoga Basin of Uganda [17] identified Oyam as one of the districts with the highest prevalence of PC. The elevated prevalence was attributed to low latrine coverage (below 50%), poor latrine conditions, limited knowledge of transmission and control, and the widespread use of free-range husbandry systems. A decade later, although latrine coverage in Oyam District has improved, the quality of facilities remains poor. The present study therefore aimed to assess the impact of increased latrine coverage on the prevalence of PC in pigs and to identify associated risk factors, using both lingual examination and antigen ELISA under the prevailing local conditions.

## 2. Materials and methods

### 2.1. Ethics statement

The study protocol was approved by Gulu University Research Ethics Committee (No: GUREC-2022–457). Trained research assistants were involved in farm sampling and data collection instruments were pre-tested before commencement of the study. The purpose of the study was explained to all study participants before including them in the study. A written consent was obtained from each farmer. Only farmers that consented were included in the study. Pigs identified for sampling were handled by trained veterinarians during sample collection to minimize distress. In addition, the identity of the respondents was kept confidential.

### 2.2. Study area

**Fig 1:**
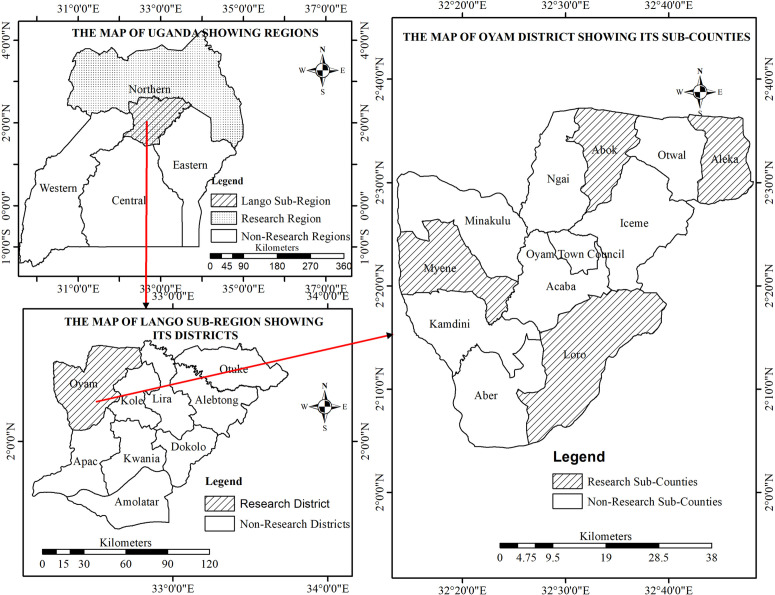
Map of Oyam District indicating the Sub-counties studied. Map of Uganda showing the location of Oyam District in the northern region (top left) and Lango Sub-region showing the location of Oyam district (below). On the right is Oyam district showing the studied sub-counties. These maps were created using ArcGIS with Ugandan administrative boundary shapefiles sourced from DIVA-GIS (https://diva-gis.org/data.html). DIVA-GIS provides open-access geographic data, and their terms of use are compatible with the CC BY 4.0 license.

The study was carried out in Oyam district, Northern Uganda (Fig 1). The district has 12 Sub - counties (Loro, Aber, Kamdini, Myene, Minakulu, Acaba, Iceme, Otwal, Aleka, Abok and Ngai) including Oyam Town Council. Physically, the district lies between latitude 2° 0‟N, 2°7”N and longitude 32° 2”E, 32°10”E. The district has a total area of 2,207km². Of these, 2% is under open swamps and water while 1% is under forests leaving 2,140.4km² of land area (97%) for both human settlement and agriculture.

Oyam district receives rainfall twice a year with one long dry season which favors crop and livestock production during the two rainy seasons. Among the major crops grown include maize, cassava, sweet potatoes and pig weed (Amaranthus) that are edible to both humans and pigs. The pig population in Oyam District is estimated at 28,350 [18], and pigs are widely distributed across the district, with most households keeping 2–5 pigs under free-range or tethering systems, thereby exposing them to *T. solium* infection.

### 2.3. Sampling and data collection

A cross-sectional study was carried out in Oyam district between January to March 2020 where a total of 394 pigs were included based on the formula of Yamane et al. [19]. A multistage sampling technique was used to select 4 sub-counties randomly out of the 12 sub-counties that make up Oyam district in the first stage, followed by 8 parishes (two per sub-county) in the second stage, while 36 villages were randomly selected at stage three and 197 pig keeping households were selected by stratified random sampling with the stratum being husbandry system in the last stage. This was done while ensuring that the number of households included per parish and village represented at least 30% of the pig keeping households. Pigs from the sampled households were then categorized by husbandry system, age and sex. A maximum of two pigs were randomly sampled per household for those with more than 2 pigs while for those with two or less pigs, all were considered in the study. Under three months pigs were excluded from the study as this age bracket was not actively involved in feeding by free range but survives by suckling the dams’ milk, thus, less likely to be infected [20]. Pregnant sows were also excluded as most of the farmers could not allow the researchers to touch them in fear of causing unnecessary distress to them.

#### 2.2.2. Porcine cysticercosis survey: Lingual examination for *T. solium* cysticerci in pigs.

All pigs were examined for the presence or absence of *T. solium* cysts on the ventral side of the tongue. The upper jaw was secured with one end piece of a rope, while the other end was fastened upwards to keep the mouth open. Using a piece of cotton cloth, the tongue was gently gripped and pulled out for examination as described by Kungu et al. [21].

#### 2.2.2. Porcine cysticercosis survey: detecting circulating *T. solium* antigens.

From each pig, 5 ml of blood was collected from the anterior vena cava into plain vaccutainer tubes and transported to the laboratory in a cool box. The samples were allowed to clot at approximately 4 °C. Serum was obtained by centrifuging the clotted blood at 2,500 rpm for 20 minutes. The serum was then aspirated using a sterile disposable pipette, transferred into cryo-tubes labeled with individual pig code, and stored at -80 °C for subsequent detection of circulating *T. solium* antigens using ELISA. A commercial ELISA assay kit for cysticercosis Antigen (Ag) detection (applied Diagnostics, Tumuhout, Belgium) was used to detect circulating *T. solium* antigens in serum samples according to the manufacturer’s guidelines (available at https://apdiagroup.com/we-sell/elisa/elisa-apdia/cysticercosis-antigen/).

#### 2.2.3. Administration of questionnaires.

A structured questionnaire was administered to each pig owner to assess the risk factors for porcine cysticercosis in the study area. The questionnaire captured data on pig management practices, pork consumption, pig breed, knowledge of the transmission of *T. solium* and porcine cysticercosis, as well as possession of latrines and household sanitation. Knowledge of transmission was assessed using a Likert scale. Farmers who responded with “agree” or “strongly agree” were considered knowledgeable, while those who responded with “disagree” or “strongly disagree” were classified as not knowledgeable. Household hygiene was assessed as either good or poor by direct observation of indicators of household hygiene such as possession of handwashing facilities among others. Farmers were also scored according to their responses to hygiene-related questions in the questionnaire.

### 2.4. Statistical analysis

Data were entered and coded in excel, then analyzed using SPSS version 16.0. Prevalence of *T. solium* cysticercosis was calculated using the formula below:


$$p=\left(\frac{d}{n}\right)*\text{1}\text{0}\text{0}\;$$

Where; **P** = the prevalence; **d** = number of samples that tested positive; **n** = the total number of samples tested. To evaluate changes in prevalence compared to the 2010 report, we calculated the difference between the prevalence reported by Nshada et al. [17] and the current study’s prevalence from lingual examination.

#### Risk factor and PC latrine possession relationship analysis.

Initial screening of potential risk factors was done using chi-square tests. Variables significantly associated with infection (*p* ≤ 0.05) were included in bivariable logistic regression models, and subsequently in multivariable model, to identify independent predictors of PC prevalence. Adjusted odds ratios (AORs) with 95% confidence intervals were calculated to determine the presence and strength of associations. History of deworming and county of origin were initially included in the model as potential confounders. However, only county of origin resulted in significant change in the odds ratios of the variables (grazing system, age, sex, breed, Hygiene, latrine presence, location and pork consumption) and was therefore maintained in the final model. To assess the association between latrine possession and PC, household hygiene, pork consumption, and farmers’ knowledge on transmission were included in the model as confounders, based on the 10% change-in-estimate rule [22]. Each variable resulted in more than a 10% change in the odds ratio for latrine possession when included. All analyses were performed at a 5% significance level.

## 3. Results

### 3.1. Demographic characteristics of the pigs and the farmers

Data on the pigs’ and farmers’ demographic characteristics, along with PC prevalence, are presented in Table 1. The majority of pigs were crossbreeds (229/396; 58%), followed by exotic breeds (119/396; 30%). More females (219/396; 55%) were examined than males (176/396; 44%), as farmers generally kept fewer males for breeding purposes. Most pigs were 8–12 months old (196/396; 49%).

**Table 1:** Porcine cysticercosis infection in pigs of different age groups, sex, breed, and husbandry system in Oyam district.

Variable	Category	n	Number of cysts	Prevalence	df	X2	P-value
Age	3-7 months	55	10	18	2	25.6	<0.001
	8-12 months	196	52	27			
	Above 12 months	145	71	49			
Sex	Male	176	66	38	1	2.2	0.086
	Female	219	67	31			
Husbandry system	Free range	150	71	47	2	22.1	<0.001
	Tethering	175	48	27			
	Semi-intensive	71	14	20			
Breed	Exotic	119	44	37	2	18.7	<0.001
	Cross	229	61	27			
	Local	48	28	58			
Presence of latrines	Yes	295	105	36	1	2.08	0.092
	No	101	28	28			
Knowledge of farmers	Good	306	111	36	1	4.36	0.042
	Poor	90	22	24			
Hygiene	Good	224	59	26	1	12.14	<0.001
	Poor	174	74	43			
Location/Sub-county	Aleka	100	21	21	3	16.02	<0.001
	Abok	107	36	34			
	Myene	70	21	30			
	Loro	119	55	46			
Pork consumption	Yes	307	111	36	1	4.05	0.055
	No	89	22	25			
Location/County	Oyam North	207	57	28	1	7.12	0.008
	Oyam South	189	78	41			
Deworming history	Yes	168	53	49	1	2.68	0.06
	No	228	55	51			

n = Number sampled per stratum.

Those with primary education made up 26%, followed by those with no formal education (18%), secondary education (16%), and tertiary education (5%). In Loro Sub County 85% of farmers had latrines, while overall latrine coverage in the district was 74%. Additionally, 77% of farmers in Oyam district demonstrated good knowledge of PC transmission.

### 3.2. Prevalence of PC in Oyam district

The overall prevalence of porcine cysticercosis (PC) in pigs in Oyam district was 27% by Lingual examination and 33% by ELISA. The prevalence of PC increased by 17% compared to the 9.4% reported over ten years ago by Nshada et al. (2010). Households with poor hygiene had the highest proportion of infected pigs (43%) compared to households that maintained proper hygiene (26%), while 58% of pigs of the local breed were found to have cysticerci. Infection rates were also higher in older (58%) and those kept under free-range systems (47%). Oyam South had a higher prevalence of PC (41%) than Oyam North (28%). Among the sub-counties, the highest prevalence was observed in Loro 55/119(46%), followed by Abok 36/107(34%) (Table 1).

### 3.3. Risk factors associated with PC in Oyam district

Multivariate logistic regression analysis revealed significant associations between PC prevalence and breed, age, husbandry system, pork consumption and household hygiene (Table 2). Local breeds were 3 times (OR=3.012; 95%CI = 1.26-7.16; p = 0.013) more likely to get infected compared to exotic and cross breeds. Pigs under 12 months of age had significantly lower odds of infection (OR=0.142; 95% CI: 0.059-0.341; p ≤ 0.001) and (OR=0.204; 95%CI: 0.084-0.495; p ≤ 0.001) than adults. Free-range and tethered pigs were almost 6 and 9 times (OR=5.71; 95% CI:1.79-18.20; p = 0.003) more likely, respectively, to be infected of PC compared to those reared under the intensive and semi-intensive systems. Pigs reared in households with poor hygiene were found to be 3 times (OR=3.407; 95% CI: 1.914-6.065; p ≤ 0.001) more likely to be infected of PC than the pigs reared in households that observed good personal hygiene and sanitation. Latrine possession was not significantly associated with infection risk.

**Table 2:** Risk factors associated with porcine cysticercosis infections in Oyam district.

Categorical Variables Considered		Frequency						
			cOR	95% CI	P-value	aOR	95% CI	P-value
Grazing system	Semi-intensive	150	1					
	Tethering	175	3.861	1.951-7.640	<0.01	9.559	3.754-24.342	≤0.01
	Free range	68	1.645	0.826-3.276	0.156	5.717	1.796-18.200	**0.003**
Sub-county	Aleka	100	1					
	Abok	107	1.874	1.010-3.479	0.047	1.936	1.022-3.670	**0.043**
	Myene	70	1.519	0.757-3.049	0.239	1.434	0.700-2.935	0.325
	Loro	119	3.047	1.681-5.523	<0.01	3.385	1.823-6.283	≤**0.01**
Age	Above 12 months	55	1					
	8-12 months	196	0.225	0.106-0.481	<0.01	0.142	0.059-0.341	≤**0.01**
	3-7 months	145	0.376	0.239-0.591	<0.01	0.204	0.084-0.495	≤**0.01**
Breed	Exotic	119	1					
	Cross	229	0.611	0.381-0.978	0.040	0.932	0.440-1.975	0.855
	Local	48	2.302	1.163-4.558	0.017	3.012	1.266-7.168	**0.013**
Sex	Male	176	0.728	0.479-1.105	0.136			
	Female	220	1					
Knowledge of how pigs get infected	Has poor knowledge	90	1					NS
	Has good knowledge	306	0.553	0.324-0.942	0.029			
Hygiene	Poor	224	2.113	1.386-3.224	<0.01	3.407	1.914-6.065	≤**0.01**
	Good	172	1					
Pork consumption	Eats pork	307	1					
	Does not eat pork	89	0.564	0.330-0.962	0.036	0.327	0.159-0.671	**0.002**
Latrine presence	Latrine available	295	0.718	0.439-1.175	0.718			NS
	Latrine missing	101	1					
Location	Oyam North	207	1					
	Oyam South	189	1.687	1.110-2.565	0.014	3.528	1.703-7.310	**0.001**

cOR: Crude odds ratio; aOR: adjusted odds ratio.

## 4. Discussion

Porcine cysticercosis remains a major problem to pig farmers globally; therefore, in-depth understanding of the occurrence of porcine cysticercosis in our communities is an important prerequisite to achieving sustainable control of infections in pigs. The overall prevalence of porcine cysticercosis in Oyam was 27.02% based on lingual examination and 33.33% by antigen ELISA (ag-ELISA). These figures are higher than previously reported rates from other districts in Uganda, for example, 15.8% in Mukono, Masaka, Kamuli and Kampala [21], 8.6% in Mukono and Kaliro [23], and 25.7% in the L. Victoria crescent [24]. However, the 33% prevalence detected by ELISA falls within the range reported in other endemic areas, such as South Africa (33%) [7], Angonia District in Mozambique (34.9%) [15], and in Zambia (57%) [25].

The current study finding represents a marked increase from the 9.4% prevalence reported in 2010 by Nsadha et al. [17] suggesting that pigs in Oyam District are highly exposed to *T. solium* eggs, likely as a result of substantial environmental contamination. In Kenya, Chege and colleagues observed a significant decrease in PC prevalence with increased latrine coverage and general improvements in hygiene and sanitation following a public health campaign [26]. This suggests that improvements in latrine coverage alone may not be sufficient to achieve a significant reduction in PC prevalence in endemic areas.

We found a significant association between the prevalence of PC and location. Loro Sub County which is a little more urbanized than Abok, Aleka and Myene and hence the highest population of people consuming pork had the highest infection rates. Over consumption of infected pork by humans in urban areas has been linked to increased chances of ingestion of *T. solium* cysts which develop into mature tape worms whose eggs are passed in human feces and can later be accessed by pigs [1]. Lack of official slaughterhouses in many areas in Uganda makes pork inspection difficult thus allowing infected pork to enter the food chain without inspection [27]. Most studies have identified the presence of pigs and high pork consumption as significant risk factors for the introduction of the disease in urban areas [28]. Uganda ranks among the largest pork consumers in sub-Saharan Africa [12], a trend attributed primarily to population growth, urbanization, and rising household incomes [29].

Free-range and tethering pig management systems were significantly associated with porcine cysticercosis in Oyam District. Pigs reared under these two systems had higher infection rates compared to those raised under semi-intensive systems. In Oyam, tethered pigs are often released in the evenings to scavenge for food in the neighborhoods, similar to free-range pigs, which explains the higher infection rates observed in these systems. In many rural communities, traditional pig husbandry practices such as free-range rearing allow pigs access to human fecal material, thereby sustaining transmission of porcine cysticercosis [5,30]. Free range system of pig management was identified as a significant risk factor for porcine cysticercosis where there is unlimited access to *T. solium* eggs by pigs from human carriers [1]. Pigs may be infected by either ingestion of feces, soil, feed stuff and water contaminated with *T. solium* eggs [31,32].

The prevalence of porcine cysticercosis (PC) increased with pig age category, with pigs over 12 months having a higher likelihood of infection compared to younger age groups. Although PC can be detected in very young pigs by Ag-ELISA as early as two weeks post-infection [27], older pigs remain at higher risk due to longer exposure periods [33,34]. Younger pigs benefit from maternal antibodies, which gradually wane in piglets born to infected sows, rendering them susceptible later [15].

Pig breed had a significant association with the prevalence of PC. Local breeds of pigs showed the highest prevalence of PC compared to exotic and cross breeds. Conversely, in the Eastern, Southern and Western provinces of Zambia, cross bred pigs were 7 times more likely to have had cysticercosis than the Nsenga (dwarf local) and exotic breeds as determined by ag-ELISA suggesting that pigs of different breeds may display different susceptibility to cysticercosis [35].

Pigs reared in households with poor hygiene had higher infection rates compared to those in households with good hygiene. Although many people use toilets, they often neglect to wash their hands thoroughly with water and soap afterward, increasing the risk of auto-infection. In addition, consumption of unboiled or untreated water contaminated with *T. solium* eggs can lead to ingestion [30], and eating unwashed fruits and vegetables can also contribute to transmission [4]. Poor sanitation, including open defecation and latrines in poor condition that allow pigs access to human feces, further increases the likelihood of porcine cysticercosis [36].

Although 27% of the households did not have pit latrines, there was no significant association between PC and possession of pit latrines in Oyam District. Previous studies identified the absence of pit latrines as a risk factor for PC [1,17,37,38]. In Angonia District, Mozambique, a high prevalence of PC (34.9%) was reported despite most households having pit latrines. Similarly, a study in Cameroon found no statistically significant difference in the prevalence of cysticercosis among pigs raised in households with or without latrines [15]. In Tanzania, Braae et al. [16] demonstrated that completely open latrines conferred a risk comparable to the absence of latrines in increasing the prevalence of PC. In Uganda, where over 80% of the population relies on ordinary pit latrines, many of which are poorly maintained, the risk of transmission may be attributable to inadequate latrine conditions and inconsistent utilization. For instance, household members may restrict latrine use to when they are at home, but practice open defecation while in the fields. Additionally, limited awareness and negative perceptions regarding latrine use remain prevalent in rural areas [11]. Given that *Taenia solium* eggs can be disseminated through domestic animals, human movement (e.g., via contaminated feet or footwear), and coprophagous insects [39], efforts to expand latrine coverage should be accompanied by improvements in latrine quality, community sensitization, and broader investments in sanitation infrastructure in the study area and in other settings with similar epidemiological profiles.

Most households involved in the study had good knowledge about *T. solium* and its mode of transmission (77.27%). However, there was no significant association between knowledge/awareness of PC and its prevalence in Oyam District. Studies have shown a relationship between PC awareness among farmers and infection rates [40]. In Tanzania, in the districts of Mbulu, Mpwapwa, Mbinga and Rungwe, knowledge about PC was good particularly among pig keepers across the districts where many participants had heard about the pork tapeworm and the knowledge about signs/symptoms and treatment were fair, but means of transmission and prevention measures were unknown [41]. On the other hand, in the districts of Kamuli and Hoima in Uganda, an interview was conducted among different stakeholders including pig farmers, community leaders, pig/pork traders, animal and human health assistants, senior officials from the ministries of agriculture and health and other relevant agencies at district level. The results showed differential, limited and fragmented knowledge on *T. solium* infections among stakeholders. Pig farmers, community leaders and pig/pork traders had almost no knowledge and were often confused regarding the differences existing between *T. solium* and other gastro-intestinal infections in pigs and humans [4]. Lack of awareness about PC and its transmission makes communities reluctant in ensuring proper hygiene and sanitation, pig confinement as well as practices that reduce on the spread of PC [27]. Some communities in endemic areas have misperceptions like “eating raw potatoes and cassava lead to tape worm infections in both pigs and humans” which has made the control of *T. solium* infection very difficult [42]. Some report reveals that communities are aware of the infection but are ignorant of how it can be transmitted and controlled [43]. While others found no significance difference in the prevalence in households with knowledge and those without [44]. This implies that occurrence of the infection in the community is attributed to risk factors other than awareness of the disease condition.

The sex of pigs showed no significant association with the prevalence of PC, suggesting that both male and female pigs were equally exposed to *T. solium* eggs. Similarly, a study conducted in The Gambia and Senegal reported no significant association between pig sex and the occurrence of PC [45]. In contrast, findings from Zuru, Nigeria, based on meat inspection, demonstrated a significant association between pig sex and infection with PC [46]. In the present study, all pigs reared within a given household were managed under the same husbandry system and fed from the same food sources, which may explain the lack of sex-related differences observed.

## 5. Conclusion

Porcine cysticercosis remains prevalent in pigs in Oyam District despite household latrine coverage ranging from 74% to 88.7%. The prevalence of infection increased from 9.4% reported in 2010 to 27% in the present study, based on lingual examination. Predisposing risk factors included age, breed, management system, hygiene, and location. No significant association was observed between latrine possession and PC, which may reflect substantial environmental contamination with *T. solium* eggs prior to improvements in latrine coverage or deficiencies in the quality and maintenance of the toilet facilities used in the study area.

These findings confirm that porcine cysticercosis remains a neglected zoonotic disease in Oyam District. To ensure sustainable and profitable pig production, while simultaneously reducing the public health burden, it is recommended that farmers adopt feasible control measures. Such measures include regular deworming of pigs with effective anthelmintics, improved husbandry practices, and the maintenance of proper household hygiene and biosecurity.

## Supporting information

S1 Data Dataset used to generate the results reported in this paper. (XLSX)

S2 Photo Poor condition of a pit latrine in one of the pig-keeping households in the study area. This field photograph illustrates the sanitation challenges observed during household visits. Photo by RW, used under CC BY 4.0.
(JPG)

S3 Photo Examination of a pig’s tongue for Taenia solium cysticerci. The white dots represent visible cysticerci detected during field inspection. Photo by RW, used under CC BY 4.0.
(JPG)
