# Supplementary figures and images for: Prevalence and risk factors associated with Taenia solium cysticercosis in pigs in Oyam district, Uganda
Source: PLoS Negl Trop Dis. 2025 Dec 2;19(12):e0013776. doi: 10.1371/journal.pntd.0013776 (PMC12688152; doi:10.1371/journal.pntd.0013776)

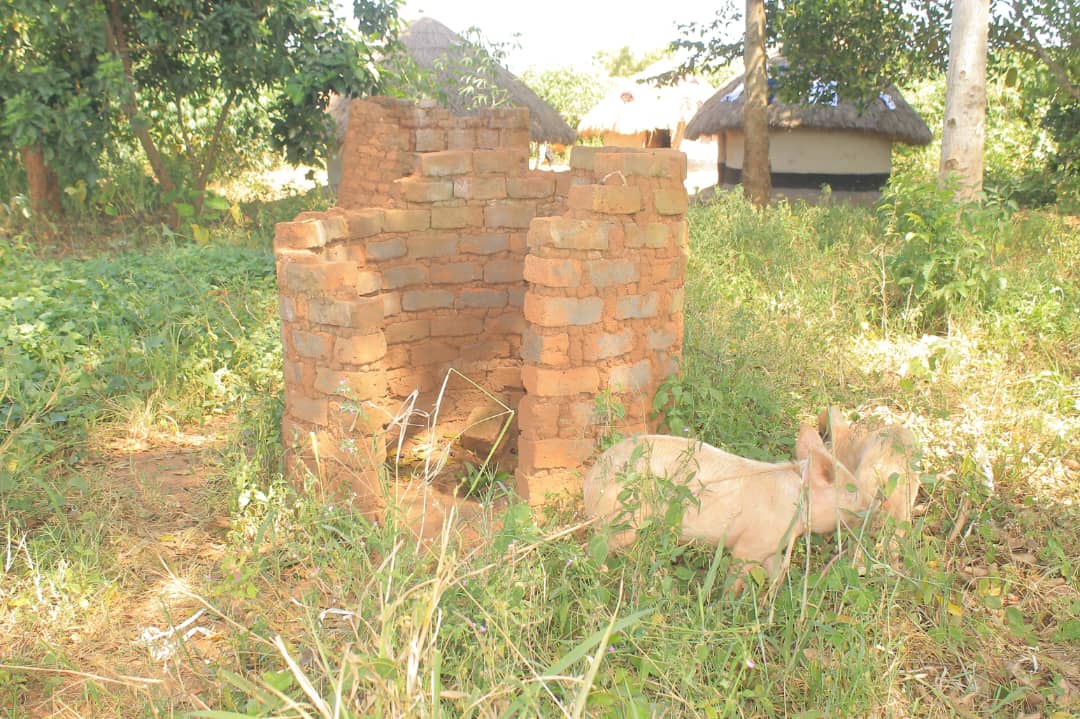

Supplement: S2 Photo — This field photograph illustrates the sanitation challenges observed during household visits. Photo by RW, used under CC BY 4.0. (JPG) [file pntd.0013776.s002.jpg]

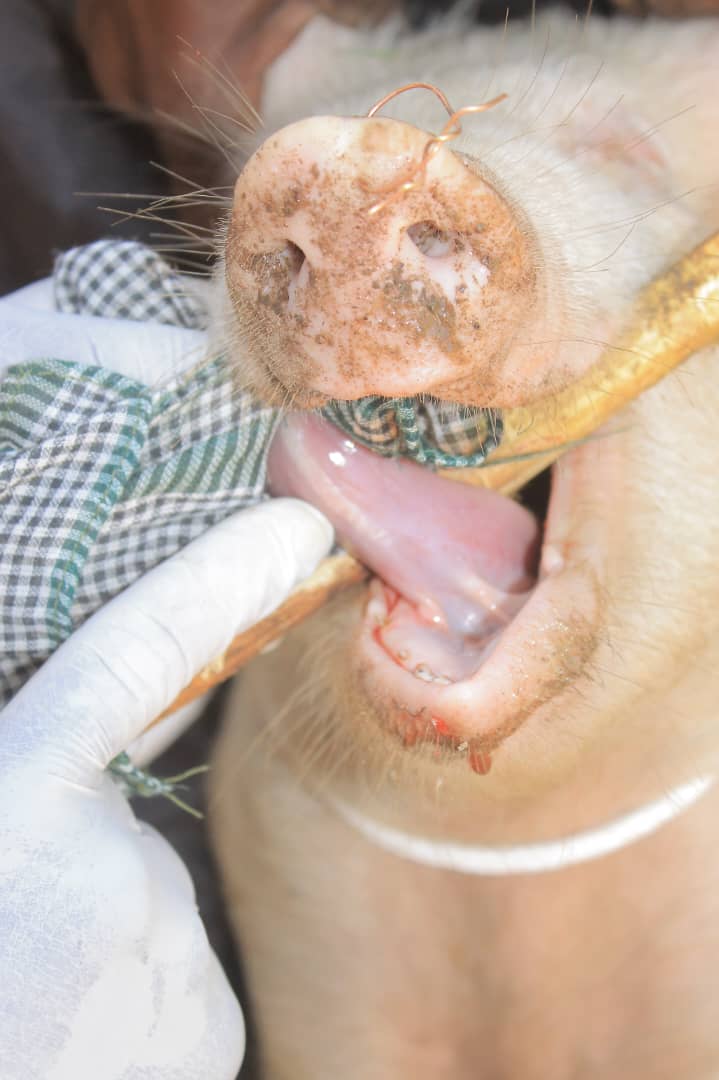

Supplement: S3 Photo — The white dots represent visible cysticerci detected during field inspection. Photo by RW, used under CC BY 4.0. (JPG) [file pntd.0013776.s003.jpg]
